# Supplementary material for: Heterogeneous multi-compartmental hydrogel particles as synthetic cells for incompatible tandem reactions
Source: Nat Commun. 2017 Sep 22;8:663. doi: 10.1038/s41467-017-00757-4 (PMC5610232; doi:10.1038/s41467-017-00757-4)
Supplement: Supplementary file 1 — Supplementary Information [file 41467_2017_757_MOESM1_ESM.pdf]

Description of Supplementary Files

Title: Supplementary Information

Description: Supplementary Figures, Supplementary Table, Supplementary Methods and Supplementary References

Title: Peer Review File

Supplementary methods

Labeling of enzymes with fluorescent dyes

GOX and HRP were labeled with rhodamine B isothiocyanate (RBITC) and fluorescein isothiocyanate (FITC), respectively. As amine-reactive fluorescent dyes, the isothiocyanate of RBITC and FITC can react with the amine groups on enzymes to form covalent bonds. Typically, 4 mg mL^{-1} of enzyme (GOX and HRP) solution dissolved in bicarbonate buffer (0.1 M, pH = 8.2) and 2.5 mg mL^{-1} of dye (RBITC and FITC) solution dissolved in anhydrous DMSO were prepared. Next, 20 µL of dye solution was added to 0.5 mL of enzyme solution and mixed well. The reactions were performed at room temperature and protected from light. After 60 min, the labeled enzymes were purified using Fluorescent Dye Removal Columns (Thermo Scientific, 22858). Finally, the purified enzymes labeled with dyes were stored at 4 °C for further use. The concentration and dye/protein ratio of RBITC-GOX and FITC-HRP were determined by the extinction coefficients of 44,100 M^{-1} cm^{-1} at 280 nm (GOX), 102,000 M^{-1} cm^{-1} at 403 nm (HRP), 108,000 M^{-1} cm^{-1} at 555 nm (RBITC), and 81,000 M^{-1} cm^{-1} at 495 nm (FITC).

H_2O_2-Amplex Red colorimetric reaction for HRP activity

In the presence of H_2O_2, HRP can oxidize Amplex Red to resorufin with a strong absorbance at 560 nm, accompanied by a red color. The detection procedure for HRP activity was as follows. Briefly, 4 µL of H_2O_2 (100 mM) and 2.5 µL of Amplex Red (1 mg mL^{-1}) were added to PBS (10 mM, pH 7.0), followed by HRP. After reacting the mixture for 15 min at 37 °C, the absorption spectrum of the reaction solution was recorded using a microplate reader (Tecan, Infinite 200 PRO, Switzerland). The HRP activity was quantified by adding various amounts of HRP to the Amplex Red-H_2O_2 solution. The final volume of the reaction solution was 200 µL, and the final concentration of HRP in the reaction solution ranged from 0 to 200 µg mL^{-1}. The absorbance intensity at 560 nm was recorded using a molar extinction coefficient of $\varepsilon = 5.8 \times 10^4$ M^{-1} cm^{-1}.

TMB colorimetric assay for MNP activity

To test the peroxidase-like activity of the MNPs, 10 µL of H_2O_2 (100 mM) and 10 µL of TMB (35 mM) were first added to NaAc buffer (10 mM, pH 4.6) followed by 3 µL of MNPs (50 µg mL^{-1}). NaAc buffer was added to reach a final volume of 400 µL. After incubating the mixture for 10 min at room temperature, the absorption spectrum of the reaction solution was measured.

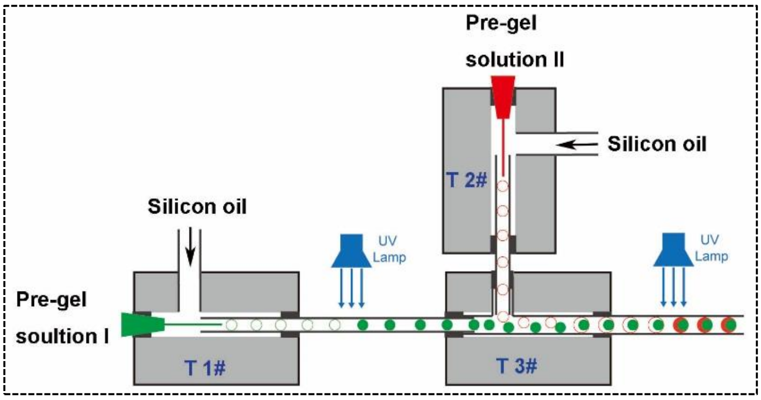

Supplementary Figure 1. Schematic representation of the fabrication process of multi-compartmental hydrogel particles.

Both the T 1# and T 2# chips were fabricated on two 40 mm × 20 mm × 2 mm PMMA plates by carving two perpendicular microchannels with a width of 1 mm and a depth of 0.50 mm to form a T-type channel using a Roland MDX-40A engraving machine. A PTFE tube (Φ0.30 × 100 mm) was inserted into the through channel of the T-type channel from one inlet, with one end of the tube reaching the joint between the perpendicular channel and the through channel. A needle with an inner diameter of 0.11 mm was also inserted into the through channel of the T-type channel from the other inlet, with the tip of the needle coaxially inserted into the PTFE tube. The two PMMA plates were sealed together using the thermal-pressing technique, and the joints were sealed with epoxy adhesive.

The T 3# chip was also fabricated on two 40 mm × 20 mm × 2 mm PMMA plates by carving a straight microchannel (W × D = 1 × 0.5 mm) using a Roland MDX-40A engraving machine. An expanding rectangle channel with a width of 1.8 mm, a depth of 0.4 mm and a length of 4 mm was carved in the upstream of the straight channel. A square PTFE tube with dimensions of 4 × 1.5 × 0.5 mm was placed in the expanding rectangle. A PTFE tube (Φ0.5 × 60 mm) was inserted into the downstream of the straight channel from the end without the expanding rectangle, with one end of the tube reaching the downstream end of the rectangle. The outlet of the PTFE tube (Φ0.3 × 500 mm) in the T 1# chip was connected to the inlet of the straight microchannel. The two PMMA plates were sealed together by the thermal-pressing technique, and the joints were sealed with epoxy adhesive.

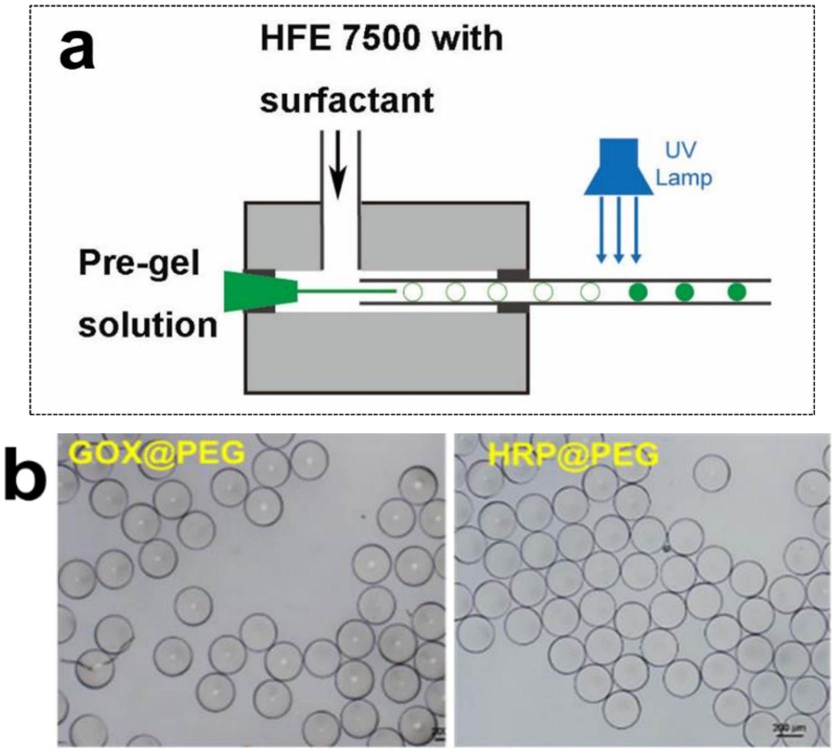

Supplementary Figure 2. (a) Schematic illustration of the fabrication of single-compartment hydrogel particles. To avoid the merging of droplets, HFE7500 with surfactants was used as the oil phase. (b) Microscopic images of poly(PEG) hydrogel-based single-compartment particles (bottom, from left to right): GOX@PEG and HRP@PEG. Scale bar: 200 μm. These three hydrogel particles were monodispersed in aqueous solution, and their diameters were almost identical.

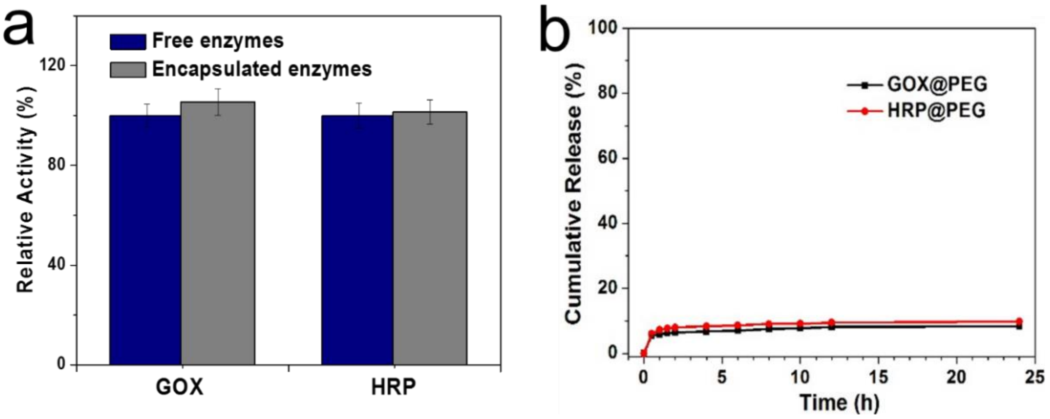

Supplementary Figure 3. (a) Relative activities of enzymes (GOX and HRP) encapsulated in poly(PEG) hydrogel (gray bars) and free enzymes (GOX and HRP) (blue bars). Identical experimental conditions were used for the reactions catalyzed by free and encapsulated enzymes (25-mM glucose as the substrate for free GOX and GOX@PEG; 2-mM H_2O_2 as the substrate for free HRP and HRP@PEG). Notably, the GOX-catalyzed glucose oxidation reaction was combined with HRP to obtain a colored product that could be quantified. (b) GOX and HRP release profiles from GOX@PEG and HRP@PEG in PBS (10 mM, pH 7.0).

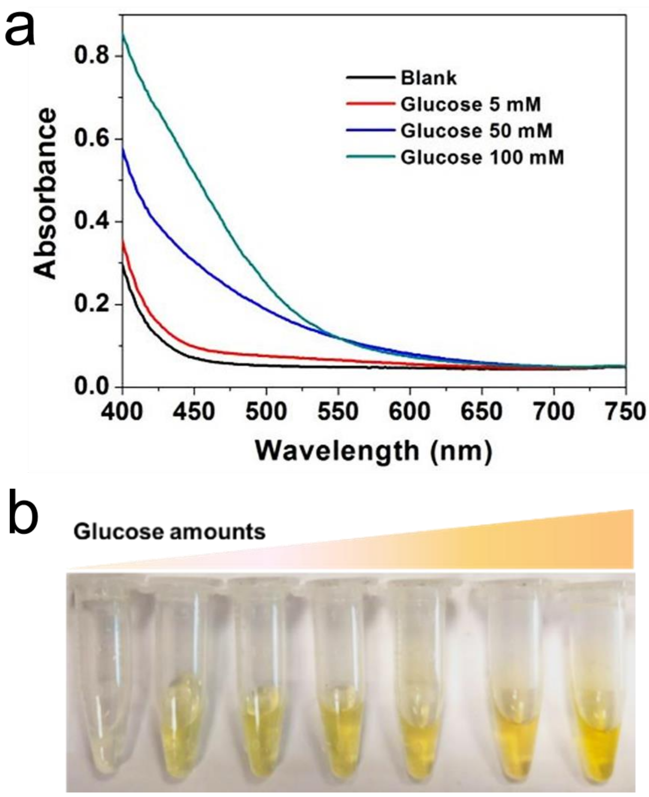

Supplementary Figure 4. Absorbance spectra (top) and visual color changes (bottom) obtained via the gluconic acid-specific assay in the presence of different concentrations of glucose ([Fe^{3+}] = 21 mM, [hydroxamine] = 125 mM, and [free GOX] = 0.25 μg mL^{-1}).

The gluconic acid-specific assay was performed according to a previous report [1], which is based on the oxidization reaction of gluconic acid with hydroxylamine and subsequently bind with Fe^{3+}, producing a red complex with a maximum absorbance at 505 nm. In this work, the detection procedure was as follows. Briefly, 200 μL of a catalytic reaction solution of GOX and glucose (in 10-mM PBS [pH 7.0] for 30 min at 37 °C) was first added to 125 μL of solution 1 (0.15-mM triethylamine in water and 5-mM ethylenediaminetetraacetic acid [EDTA]) and 12.5 μL of solution 2 (3-M NH_2OH in water) and mixed well. After reacting the mixture for 30 min at 37 °C, 62.5 μL of solution 3 (0.1-M $FeCl_3$, 1 M HCl, and 0.25-M CCl_3COOH in water) was then added to the mixture, and the reaction was allowed to proceed for an additional 5 min at 37 °C.

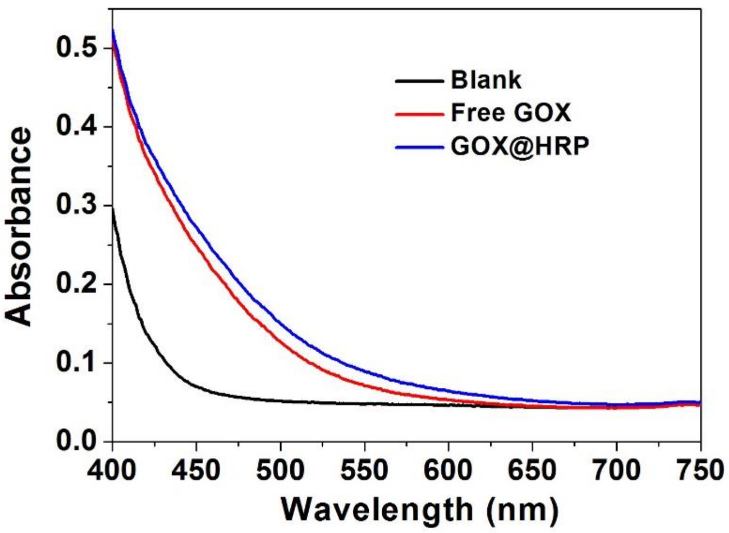

Supplementary Figure 5. Absorption spectra obtained from gluconic acid-specific assays in the absence and presence of free GOX and GOX@HRP. ([glucose] = 25 mM, [Fe^{3+}] = 21 mM, [hydroxamine] = 125 mM, and [free GOX] = GOX@HRP = 0.25 μg mL^{-1}).

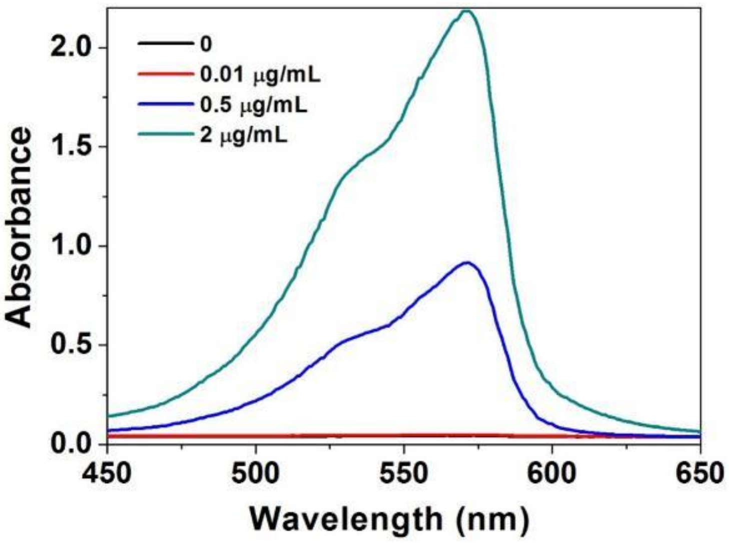

Supplementary Figure 6. Absorption spectra of resorufin produced from the H_2O_2-Amplex Red colorimetric assay in the presence of different concentrations of free GOX. Assay conditions: different concentrations of free HRP were incubated with 2 mM H_2O_2 and 15 μg mL^{-1} Amplex Red in PBS (10 mM, pH 7.0).

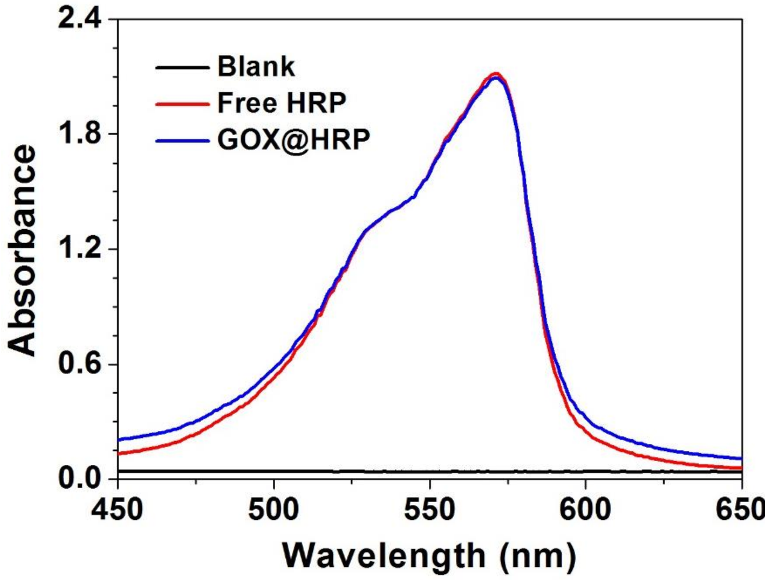

Supplementary Figure 7. Absorption spectra of resorufin produced from the H_2O_2-Amplex red assay in the absence and presence of free HRP and GOX@HRP. ([H_2O_2] = 2 mM, [Amplex Red] = 15 µg mL^{-1}, [free HRP] = 0.2 µg mL^{-1}, and GOX@HRP = 0.23 µg mL^{-1}).

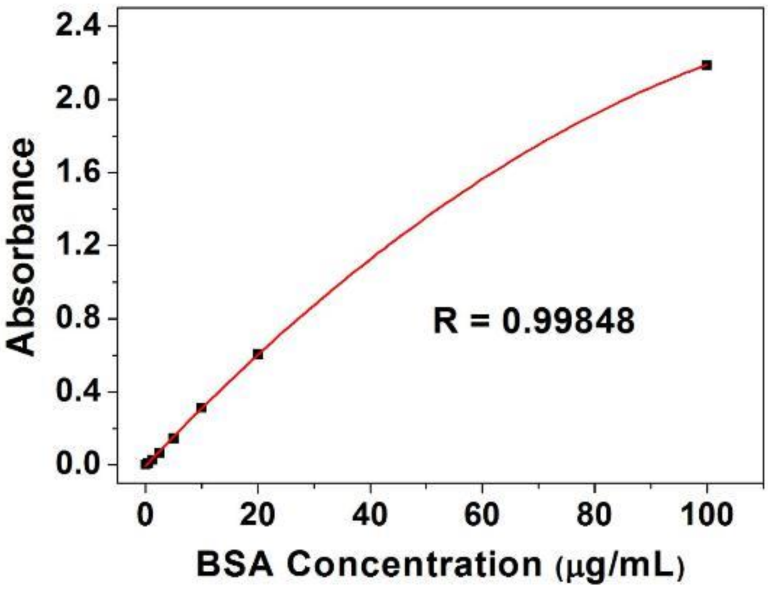

Supplementary Figure 8. Determination of the protein contents using a BCA assay. The absorbance was at 560 nm, and the spectra of the samples subjected to the BCA assay in the presence of different concentrations of bovine serum albumin (BSA) were collected to demonstrate the corresponding visual color changes.

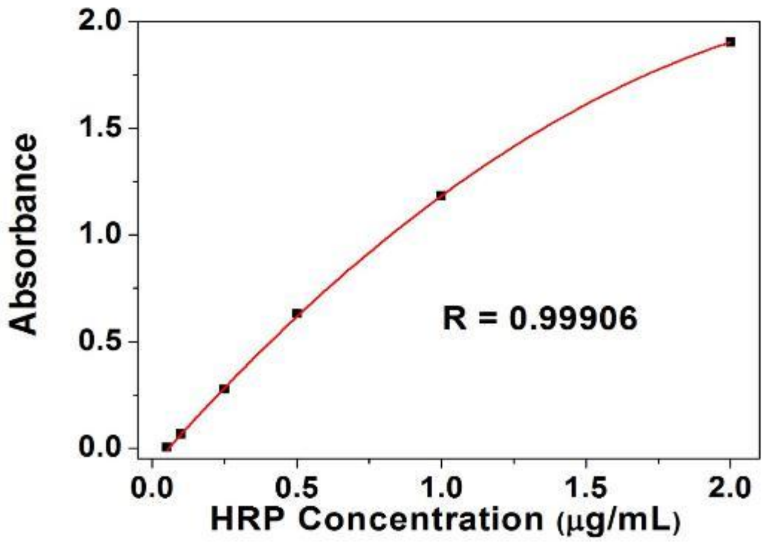

Supplementary Figure 9. Absorption spectra of resorufin at 560 nm obtained using different HRP concentrations. Assay conditions: 2 mM H_2O_2 and 15 μg mL^{-1} Amplex Red were incubated with different concentrations of HRP in PBS (10 mM, pH 7.0).

Supplementary Table 1. Kinetic data for separate GOX and HRP in GOX@HRP and free GOX and HRP

Catalyst	Substrate	*K*m / mM	*V*max / 10^{-8} M s^{-1}	*K*cat / s^{-1}
**Free GOX**	Glucose	0.643	5.417	168.482
**GOX@HRP**	Glucose	0.613	6.594	172.935
**Free HRP**	H_2O_2	0.626	5.296	388.527
**GOX@HRP**	H_2O_2	0.566	6.039	403.146

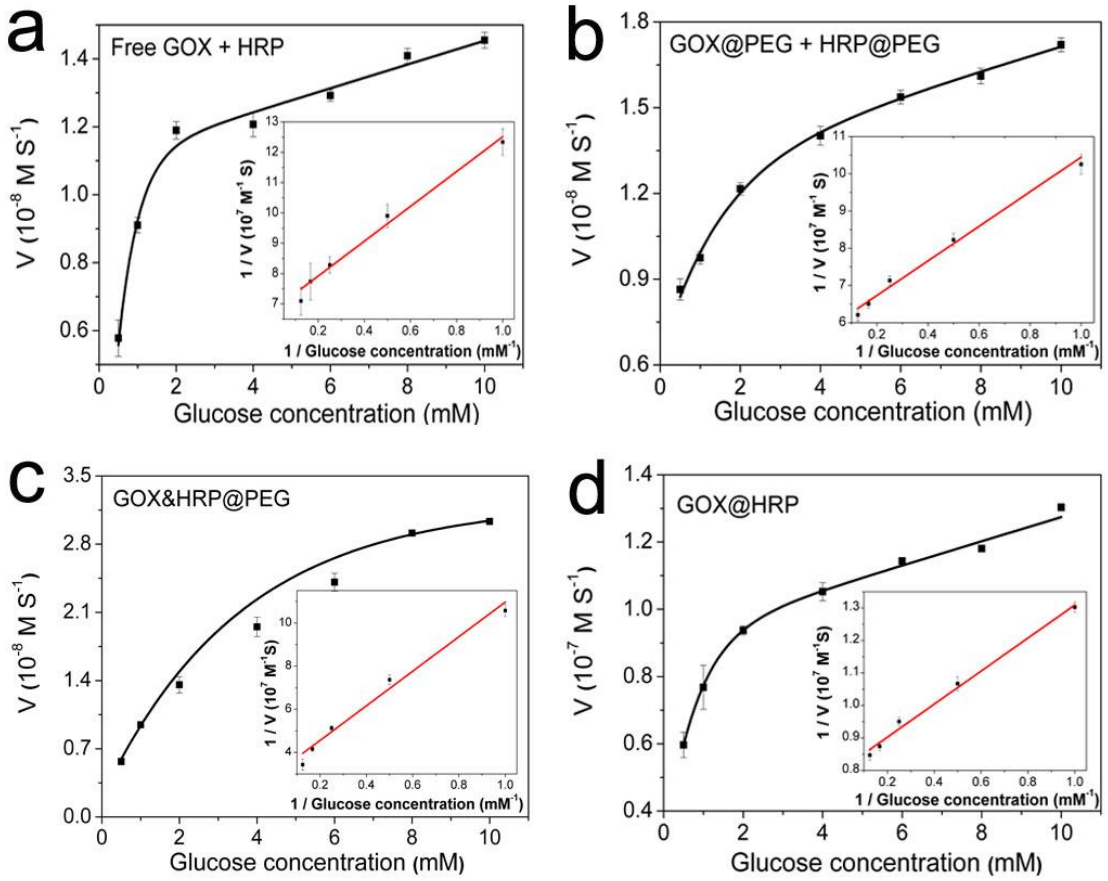

Supplementary Figure 10. Steady-state kinetic assay of tandem reactions performed using different systems: the free GOX/HRP system (a), the GOX@PEG/HRP@PEG system (b), GOX&HRP@PEG (c), and GOX@HRP (d). The insets are their corresponding double-reciprocal plots.

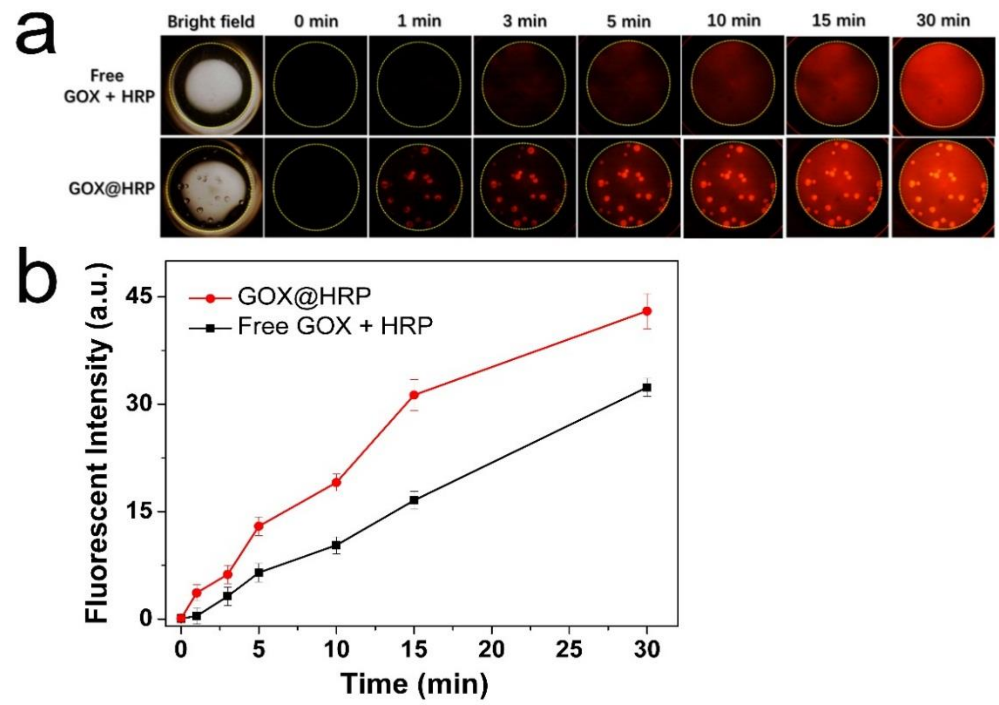

Supplementary Figure 11. Time-dependent fluorescence imaging of Amplex Red in the presence of GOX@HRP and the free GOX/HRP system (a) and their corresponding fluorescent intensities (b).

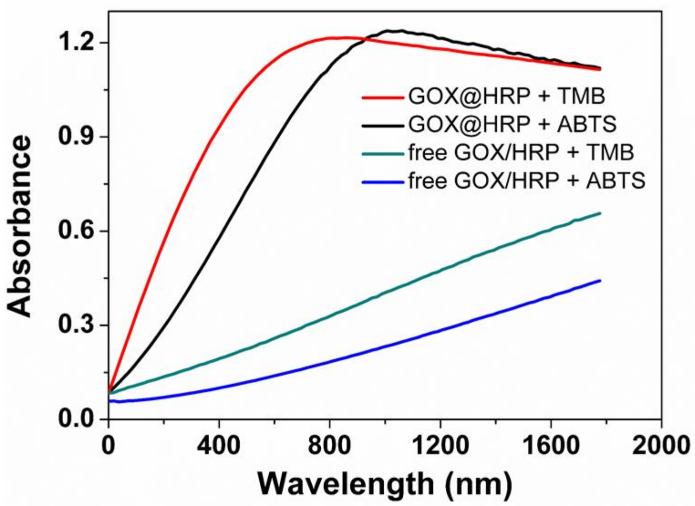

Supplementary Figure 12. Time-dependent absorbance changes as a result of the oxidation products of TMB and ABTS catalyzed by GOX@HRP and the free GOX/HRP system.

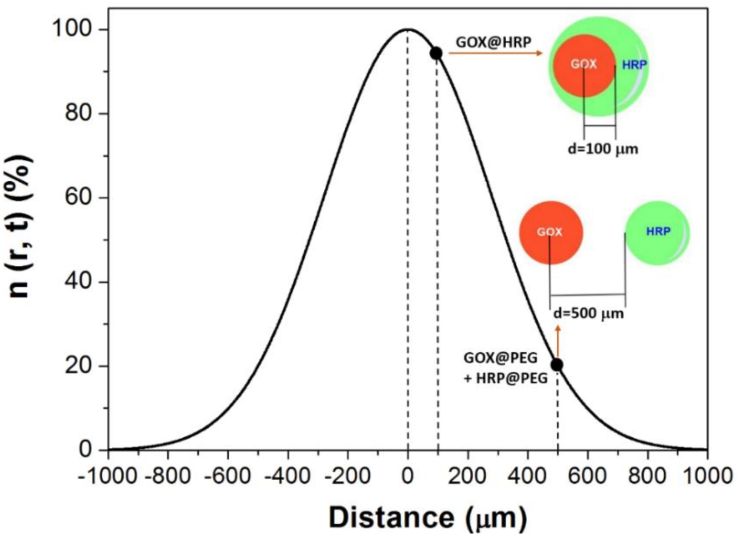

Supplementary Figure 13. H_2O_2 concentration gradient as a function of distance with following parameters: diffusion coefficient ~1000 m^2/s, *K*cat (in GOX reaction) ~300 s^{-1}, and the integration time ~100 s. The model of H_2O_2 diffusion is calculated by using Brownian motion model (Supplementary Equation-1) reported before [2,3].

$$\mathrm{n}(r,t) = \sum_{i=0}^{i=\frac{t}{\tau}-1} \frac{1}{\left(4\pi D(t-i\tau)\right)^{\frac{3}{2}}} \exp\left(-\frac{r^2}{4D(t-i\tau)}\right) \quad (\text{Supplementary 1})$$

Supplementary Equation-1: The convolution function of Brownian motion of H_2O_2 with a constant catalytic rate for a GOx/HRP pair in the given time t is described, where n (*r*, *t*) is the concentration of H_2O_2 at a distance *r* from the initial position, D is the diffusion coefficient, and τ is the average time between GOX turnovers (1/ *K*cat).

In this simulation, it was found that without dense packing of GOX and HRP (GOX@PEG + HRP@PEG system), the distance between GOX@PEG hydrogel particle (first step enzyme reaction) and HRP@PEG hydrogel particle (second step enzyme reaction) was ~500 μm. Therefore, when H_2O_2 diffused to HRP@PEG, the concentration decreased to ~20 % of the initial concentration of H_2O_2 (assume the GOX reaction occurred in the center of the core), causing limited efficiency to produce resorufin. While in GOX@HRP system, with dense packing of GOX and HRP in a hydrogel particle via microfluidics, the distance between GOX hydrogel component (core part, first step enzyme reaction) and HRP hydrogel component (shell part, second step enzyme reaction) was ~100 μm. Therefore, the H_2O_2 concentration remained ~90 % of the initial concentration of H_2O_2 to effectively trigger following HRP enzyme reaction, generating resorufin.

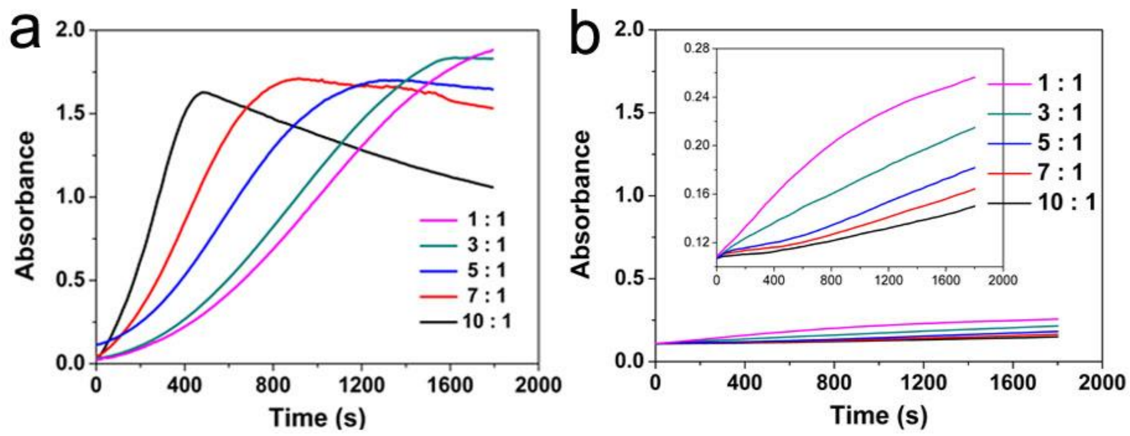

Supplementary Figure 14. Time-dependent absorbance changes as a result of the oxidation product of Amplex Red catalyzed by GOX@HRP (a) and GOX&HRP@PEG (b) with molar ratios of GOX and HRP from 1:1 to 10:1.

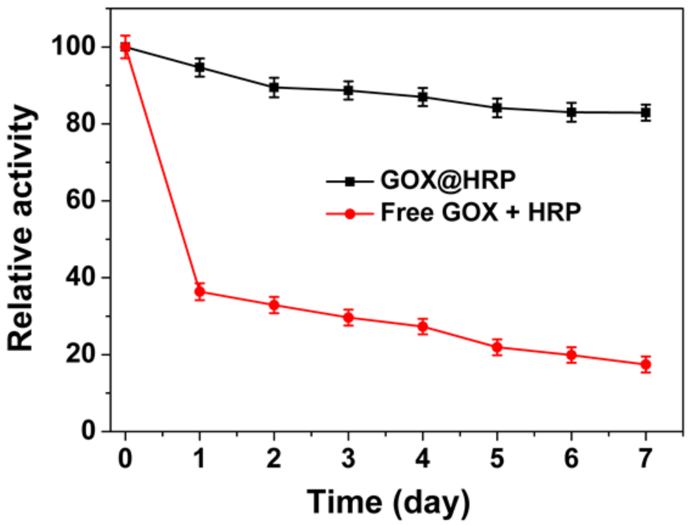

Supplementary Figure 15. Relative activity changes of GOX@HRP and the free GOX/HRP system after storage in an aqueous solution at room temperature (25 °C) for different durations.

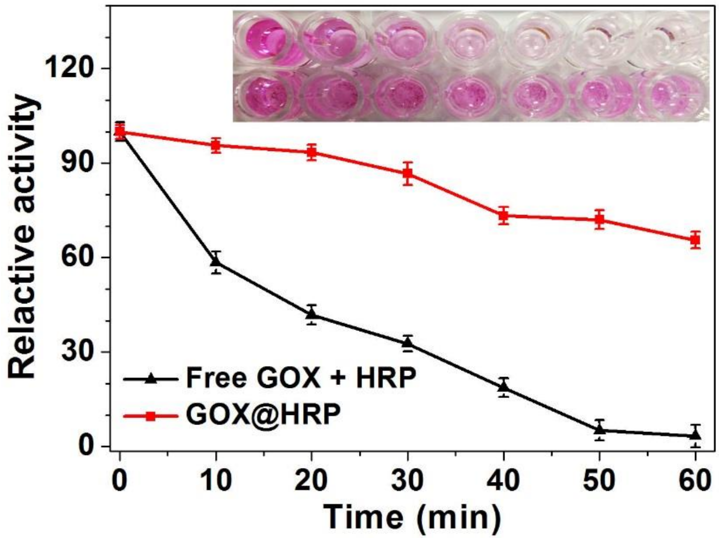

Supplementary Figure 16. Changes in the relative activities of the free GOX/HRP system and GOX@HRP after incubation at 65 °C for different durations. Inset: corresponding visual color changes.

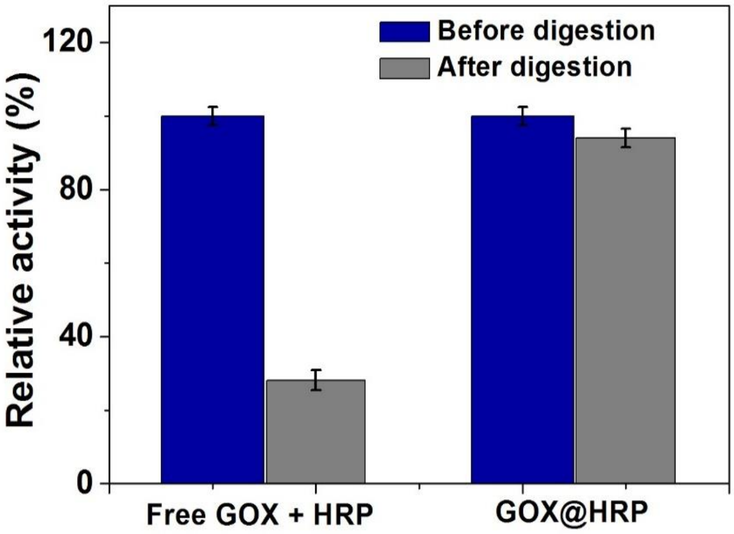

Supplementary Figure 17. Changes in the relative activities of free GOX/HRP pairs and GOX@HRP after digestion with excess trypsin (1 mg mL^{-1}) for 24 h at 37 °C.

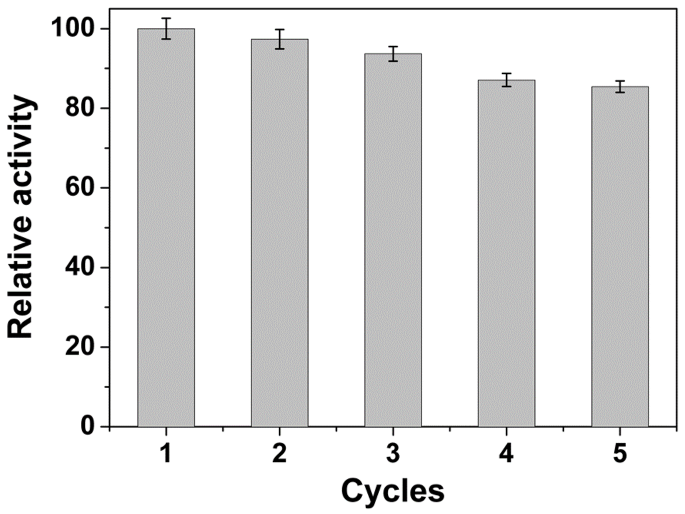

Supplementary Figure 18. Changes of relative catalytic activities of the GOX@HRP system after recycling (5 repeated experiments).

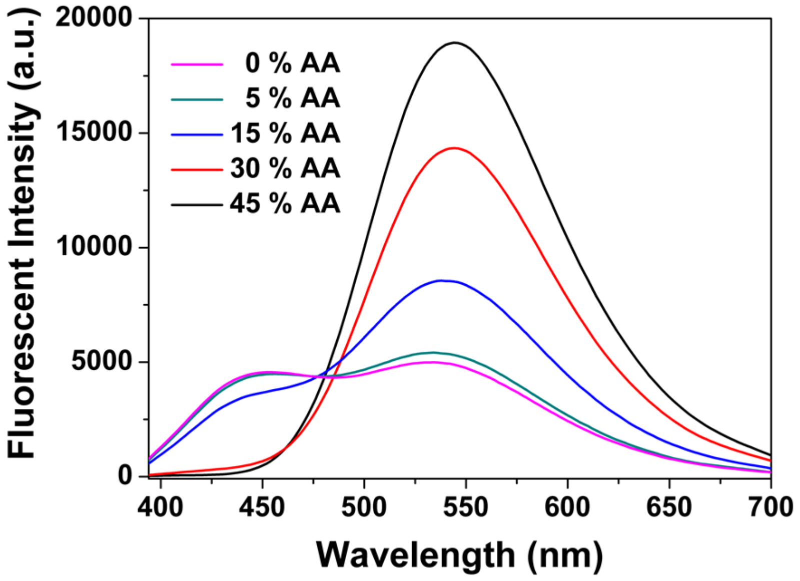

Supplementary Figure 19. Emission spectra of the LysoSensor conjugated with dextran encapsulated in poly (PEG-co-AA) hydrogels with different AA amounts (from 0 to 45 %).

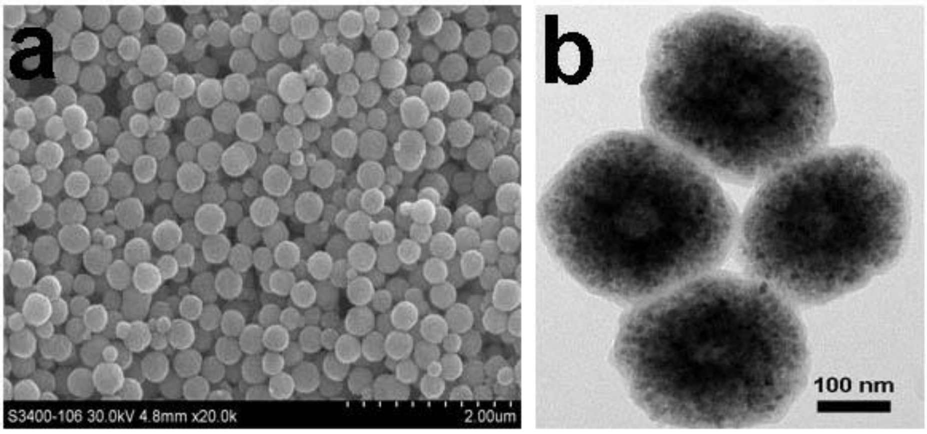

Supplementary Figure 20. SEM (a) and TEM (b) images of MNPs. The MNPs with a core/shell structure were produced according to a previous report [4]. Typically, a ferrocene solution was first prepared by dissolving 0.3 g of ferrocene in 30 mL of acetone. After intense sonication for 30 min, 1 mL of H_2O_2 was added dropwise to the above ferrocene solution and allowed to react for 15 min with stirring. Next, the resulting solution with a total volume of 50 mL was transferred to a Teflon-lined stainless steel autoclave. The solvothermal reaction was conducted for 24 h at 210 °C. The products were collected under a magnetic field after cooling to room temperature and washed several times with absolute ethanol to remove unreacted reactants. Finally, the MNPs were dispersed in ultrapure water.

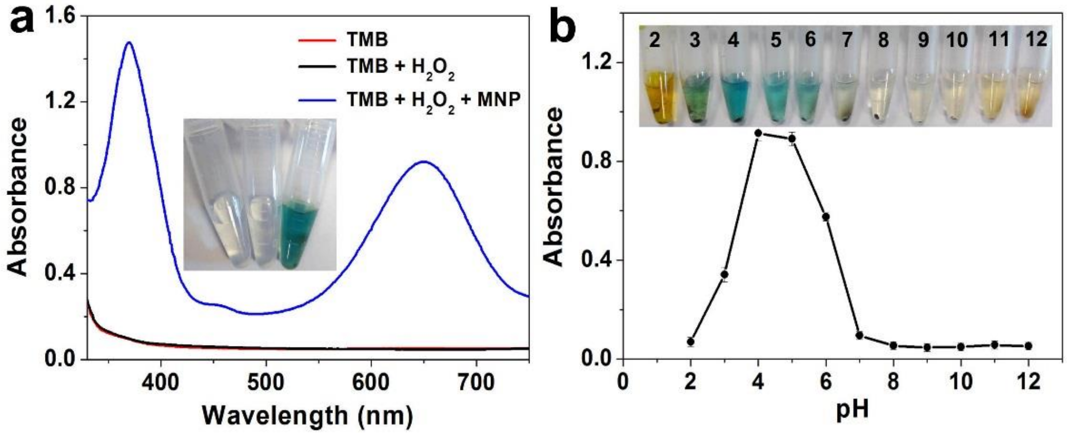

Supplementary Figure 21. Peroxidase-like activity of MNPs. (a) Absorption spectra and visual color changes of 3,3',5,5'-tetramethylbenzidine (TMB) under different conditions: control (red line); H_2O_2 (black line); and H_2O_2 and MNPs (blue line). (b) Effects of pH on the peroxidase-like activity of MNPs. ([TMB] = 43.75 µM, [H_2O_2] = 1.25 mM, and [MNP] = 50 µg mL^{-1}).

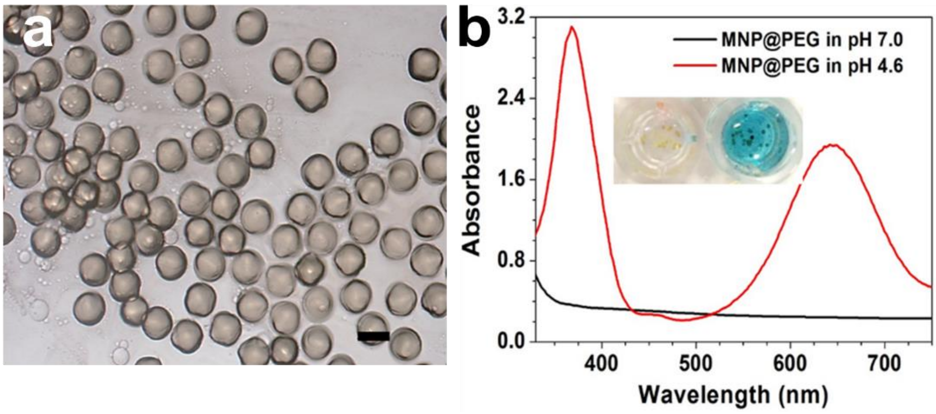

Supplementary Figure 22. (a) Microscopic image of MNP@PEG particles. Scale bar: 200 µm. (b) Absorption spectra of the TMB-H_2O_2 colorimetric assay catalyzed by MNP@PEG in PBS with pH 7.0 (black line) and pH 4.6 (red line). Inset: corresponding visual color changes of the two samples.

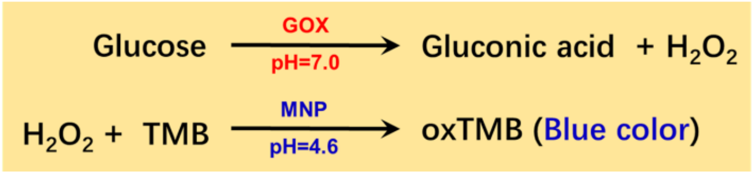

Supplementary Figure 23. Reaction equations of glucose and TMB oxidization catalyzed by GOX and MNPs to produce oxTMB. These reactions were conducted in two steps in different pH environments (7.0 and 4.6).

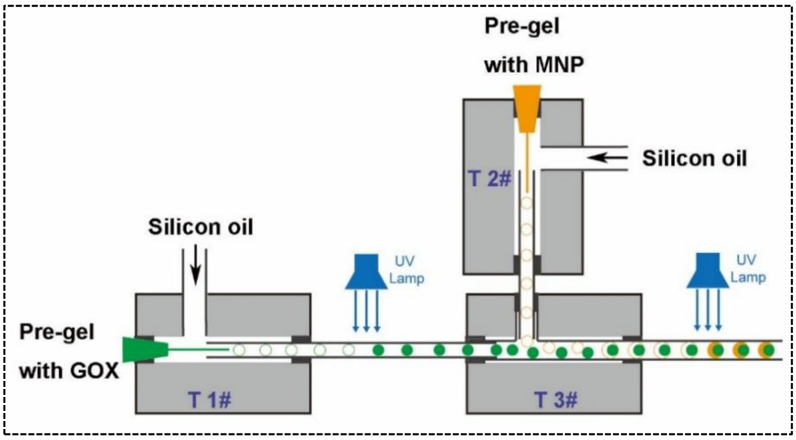

Supplementary Figure 24. Schematic illustration of the fabrication of heterogeneous multi-compartmental hydrogel particles (GOX@MNP). PEG (Mn = 575) was used as a monomer to fabricate the inner compartments, whereas the outer compartment was fabricated using PEG (Mn = 700) as the monomer and AA as the co-monomer.

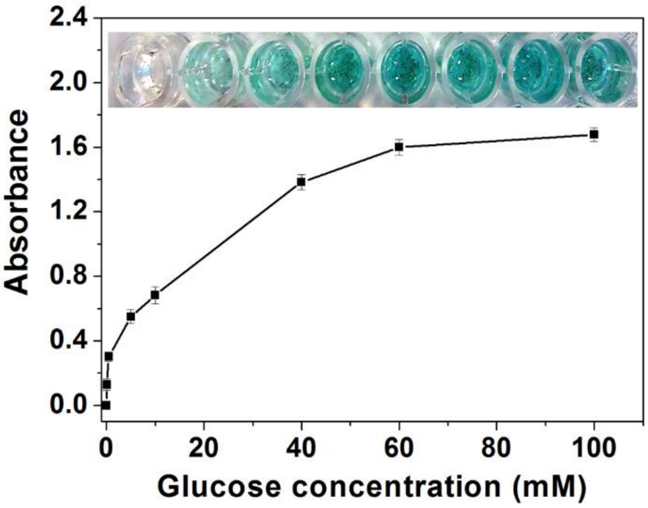

Supplementary Figure 25. Absorbance of oxTMB in the GOX@MNP-based reaction system in the presence of different concentrations of glucose. Assay conditions: 1 μg mL^{-1} GOX@MNP and 43.75 μM TMB were incubated with different concentrations of glucose in PBS (10 mM, pH 7.0) at 37 °C for 30 min.

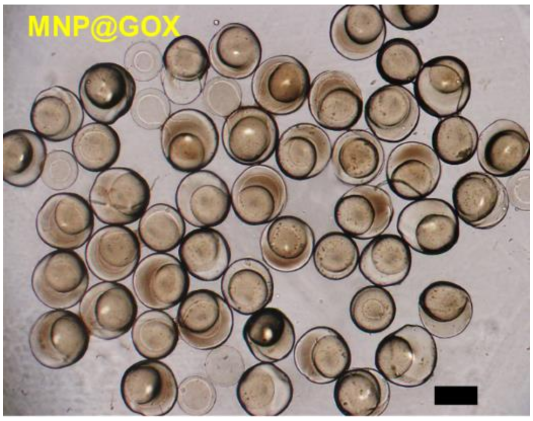

Supplementary Figure 26. Microscopic image of MNP@GOX. Scale bar: 200 μm. MNPs and GOX are separately confined in the inner and outer compartments, respectively, within the particles.

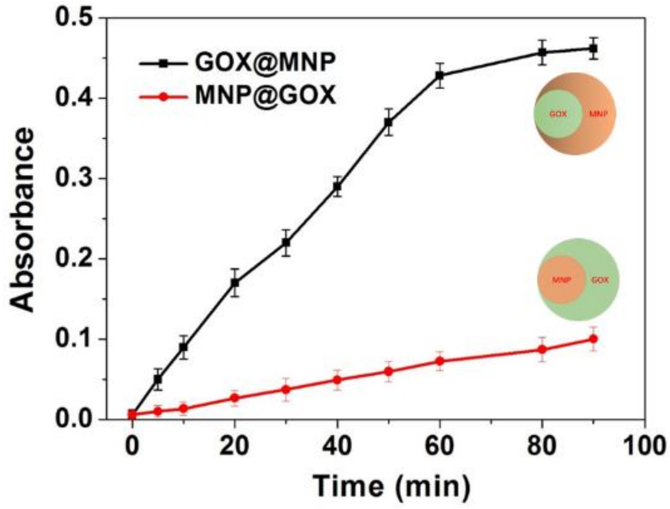

Supplementary Figure 27. Time-dependent absorbance of the oxidation product of Amplex Red at 560 nm catalyzed by the same amounts of GOX@MNP (black line) and MNP@GOX (red line) in the presence of 25 mM glucose. The reactions were conducted in PBS (10 mM, pH 7.0).

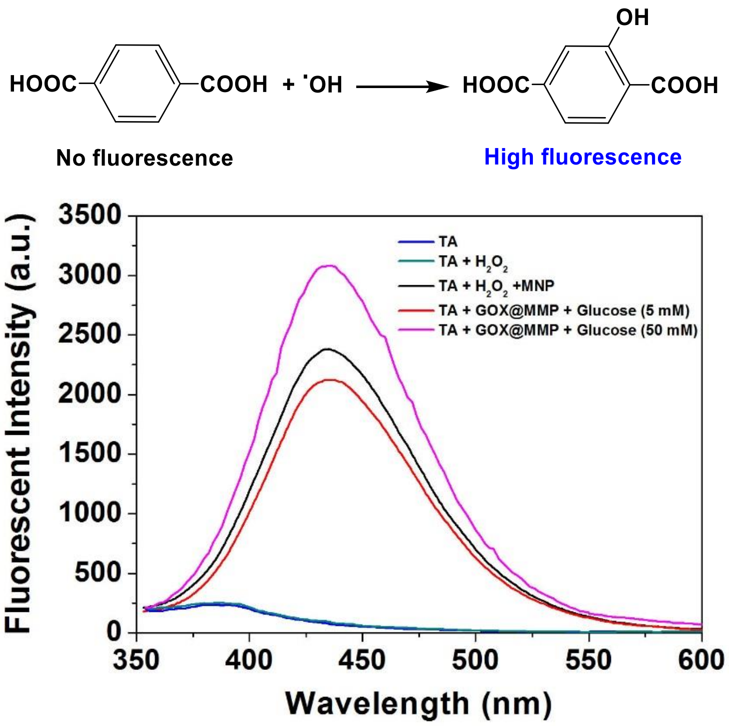

Supplementary Figure 28. Hydroxyl radicals (·OH) inducing the conversion of non-fluorescent TA to highly fluorescent 2-hydroxy TA (top) and the emission spectra of TA (bottom) under different conditions: alone, H_2O_2 only, H_2O_2 + MNP, GOX@MNP + glucose (5 mM), and GOX@MNP + glucose (50 mM). The reaction was performed in HAc-NaAc buffer (10 mM, pH 4.6) with 100 μM TA, 10 mM H_2O_2 and 50 μg of MNPs. The total volume was 200 μL.

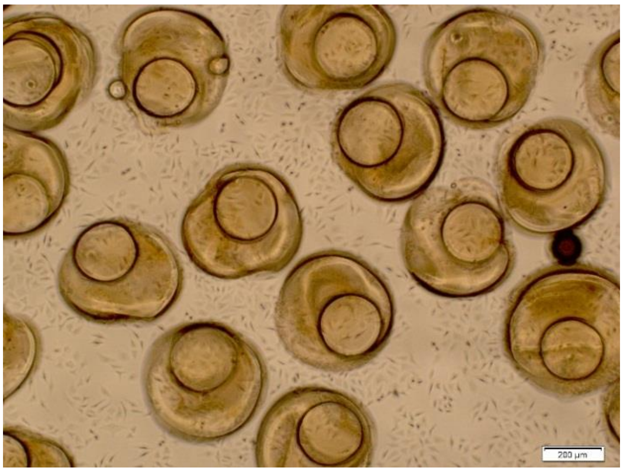

Supplementary Figure 29. Microscopic image of HeLa cells cultured with GOX@MNP.

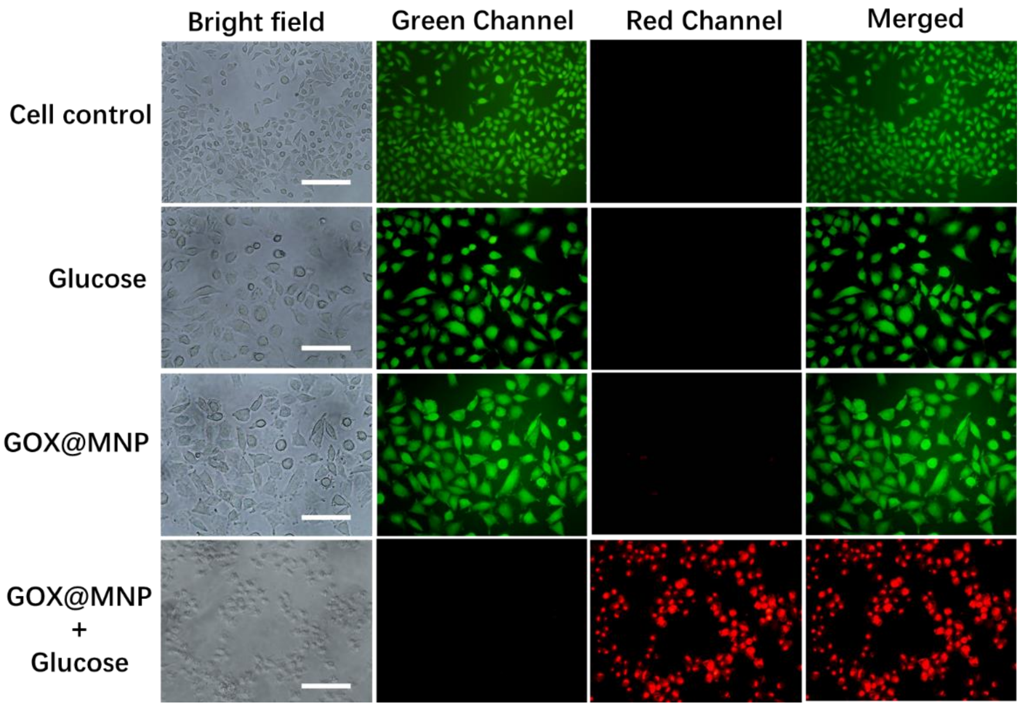

Supplementary Figure 30. Staining of HeLa cells by FDA (green fluorescence) for living cells and PI (red fluorescence) for dead cells. Microscopic fluorescence images of HeLa cells either incubated in culture medium without glucose (control) or treated with 50 mM glucose, 5 μg mL^{-1} GOX@MNP, or a mixture of 50 mM glucose and 5 μg mL^{-1} GOX@MNP. From left to right: bright field, green channel, red channel, and merged image of the FDA and PI channels for the same cells. Scale bar: 200 μm.

Supplementary references

1 Luo, W., Zhu, C., Su, S., Li, D., He, Y., Huang, Q. & Fan, C. Self-catalyzed, self-limiting growth of glucose oxidase-mimicking gold nanoparticles. *ACS Nano* **4**, 7451-7458, (2010).

2 Fu, J., Liu, M., Liu, Y., Woodbury, N. W. & Yan, H. Interenzyme substrate diffusion for an enzyme cascade organized on spatially addressable DNA nanostructures. *J. Am. Chem. Soc.* **134**, 5516-5519, (2012).

3 Wheeldon, I., Minteer, S. D., Banta, S., Barton, S. C., Atanassov, P. & Sigman, M. Substrate channelling as an approach to cascade reactions. *Nat. Chem.* **8**, 299-309, (2016).

4 Li, Q., Tang, G., Xiong, X., Cao, Y., Chen, L., Xu, F. & Tan, H. Carbon coated magnetite nanoparticles with improved water-dispersion and peroxidase-like activity for colorimetric sensing of glucose. *Sens. Actuators B-Chem.* **215**, 86-92, (2015).
